# Sub-optimal maternal gestational gain is associated with shorter leukocyte telomere length at birth in a predominantly Latinx cohort of newborns

**DOI:** 10.1186/s40748-023-00167-z

**Published:** 2023-11-03

**Authors:** Apurva Prasad, Jue Lin, Laura Jelliffe-Pawlowski, Kimberley Coleman-Phox, Larry Rand, Janet M Wojcicki

**Affiliations:** 1 Division of Pediatric Gastroenterology, Hepatology and Nutrition, Department of Pediatrics, University of California, San Francisco, USA; 2 Department of Biochemistry and Biophysics, University of California, San Francisco, USA; 3 Preterm Birth Initiative, University of California, San Francisco, USA; 4 Department of Epidemiology and Biostatistics, University of California, San Francisco, USA; 5 Department of Obstetrics, Gynecology and Reproductive Health Sciences, University of California, San Francisco, USA

## Abstract

**Objective:**

To assess in utero exposures associated with leukocyte telomere length (LTL) at birth and maternal LTL in a primarily Latinx birth cohort.

**Study design:**

Mothers and newborns were recruited postnatally before 24 h of life. Newborn LTL was collected via heelstick at birth and maternal LTL was collected postnatally. LTL was determined by quantitative PCR. Using a longitudinal design, we evaluated associations between neonatal and maternal LTL and appropriate maternal gestational gain as indicated by the American College of Obstetrics and Gynecology (ACOG).

**Result:**

Mean infant LTL was 2.02 ± 0.30 T/S (*n* = 386) and maternal LTL was 1.54 ± 0.26 T/S (*n* = 58). Independent risk factors for shorter LTL at birth included longer gestational duration (Coeff:-0.03, 95%CI: -0.05—0.01;*p* < 0.01) and maternal gestational weight gain below ACOG recommendations (Coeff:-0.10, 95%CI: -0.18 – -0.02; *p* = 0.01).

**Conclusion:**

Gestational weight gain below ACOG recommendations may adversely impact neonatal health in Latinx infants as indicated by shorter LTL at birth.

## Background

Telomeres, a biomarker of mitotic replicative history in somatic cells [45], shorten in tandem with the biological age of an organism [7]. Rate of attrition is dependent upon the presence of risk factors [53], with some studies noting the negative effects of lower socioeconomic status [11, 18], mental illness [59], smoking [63], and toxic environmental exposures [70] on leukocyte telomere length (LTL). Shorter LTL is a known risk factor for disease, including certain types of cancer [7, 41] and chronic metabolic disease include type 2 diabetes mellitus and cardiovascular disease [17, 27, 66].

There is significant LTL variability at birth [53]. Most studies have found female LTL to be longer than in males, including some of our own [39, 53, 69]. Other factors that are associated with shorter LTL at birth include more advanced parental age [12, 71] exposures to oxidative stress, including maternal smoking or secondhand smoke exposure in utero [48, 63] and the presence of maternal mental illness or stress during pregnancy [23, 64].

Previous studies have found race/ethnicity to be a significant factor impacting LTL at birth [20, 34, 39]. Most studies, however, only assess differences between participants of white or black racial background [34, 55], or only assess LTL later in childhood in diverse cohorts [49, 50]. Few studies have addressed relationships between race or ethnicity and LTL at birth or included Latinx children apart from our previous publications [10, 69]. Latinx families are one of the fastest growing [28], historically and contemporarily marginalized [2] population groups in the United States. Close to 50% of new births in the state of California are Latinx and Latinx newborns represent the largest population demographic of young children [54].

As shorter LTL at birth is associated with long-term adverse health outcomes [14, 43, 51], elucidating in utero factors that impact LTL could have major public health implications for this important population group. In this study, we evaluated the role of maternal weight gain in pregnancy on newborn LTL as excessive or inadequate weight gain in pregnancy can have short and long-term consequences including obesity and metabolic disease [9, 22].

## Methods

### Cohort and inclusion, exclusion criteria

Data were collected from the Telomere at Birth (TAB) cohort, a longitudinal study of mothers and babies recruited postnatally at UCSF Benioff and Zuckerberg San Francisco General Hospitals to assess how intrauterine exposures impact LTL. Eligibility criteria for maternal enrollment included English or Spanish speaking, no history of active illicit drug use and plans to stay in the San Francisco Bay Area for the foreseeable future. Eligibility criteria for infant enrollment included > 32 weeks gestational age, no contraindications for breastfeeding, in the newborn or intensive care nursery without any imminent surgery or chronic disease condition including possibility of HIV infection or COVID-19. All study procedures were approved by the Institutional Review Board (IRB) at the University of California, San Francisco (UCSF) and all patients signed informed consent for their and their children’s participation. There were no financial competing interests associated with this research. All methods were carried out in accordance with relevant guidelines and regulations (Declaration of Helsinki).

### Study procedures

Eligible study participants were screened via electronic medical record review in both hospitals and subsequently approached prior to 24-h postpartum when the California Genetic Newborn Screen is routinely conducted. Bilingual research coordinators explained all study procedures to potential participants. Participants were subsequently interviewed using a one-page questionnaire to collect information on exposures in pregnancy including dietary ones (specifically sugar sweetened beverage (SSB), defined as all sodas, fruit juices and sweetened teas, and 100% fruit juice intake during pregnancy), smoking/second hand smoke exposure and weight gain in pregnancy. Demographic and household information was also collected including race/ethnicity, marital status, smoking history and exposure to secondhand smoke. Self-reported medical conditions and health history were also collected including diagnoses in pregnancy such as gestational diabetes mellitus and hypertension, age of menarche and the medical record was used to confirm all diagnoses. Centers for Disease and Control (CDC) growth curves were used to calculate newborn percentiles and Z scores [36]. All data was uploaded into RedCap and analyzed using Stata 15.0 and 16.0 (StataCorp, College Station, TX, USA). and Python 3.

### Leukocyte telomere length analysis

DNA was extracted from the dried blood spots (DBS) on Whatmann 903 cards with the QIAamp DNA Investigator kit (QIAGEN cat# 56,504) and eluted in 50 ml ATE buffer. The DBS were collected between August 2016 to March 2020, stored at -80ºC and batch-extracted in November 2020. The DNA yields from DBS are too low to be accurately measured by OD260/OD280. Instead, based on our experience, we used 8 ul of extracted DNA. The values from the single-copy genes were within the standard curve, confirming that we had used the proper amount of input DNA.

DNA was stored at -80ºC and LTL assays were performed between November to December of 2020. The telomere qPCR primers were tel1b [5'-CGGTTT(GTTTGG)5GTT-3'], used at a final concentration of 100 nM, and tel2b [5'-GGCTTG(CCTTAC)5CCT-3'], used at a final concentration of 900 nM. The single-copy gene (human beta-globin) qPCR primers were hbg1 [5'-GCTTCTGACACAACTGTGTTCACTAGC-3'], used at a final concentration of 300 nM, and hbg2 [5'-CACCAACTTCATCCACGTTCACC-3'], used at a final concentration of 700 nM. The final reaction mix consisted of the following: 20 mM Tris-hydrochloride, pH 8.4; 50 mM potassium chloride; 200 μM each deoxyribonucleotide triphosphate; 1% dimethyl sulfoxide; 0.4 × SYBR green I; 22 ng Escherichia coli DNA; 0.4 Units of platinum Taq DNA polymerase (Invitrogen Inc., Carlsbad, CA), and approximately 6.6 ng of genomic DNA per 11 µl reaction. A threefold serial dilution of a commercial human genomic DNA (Sigma-Aldrich, cat#11,691,112,001) containing 26, 8.75, 2.9, 0.97, 0.324 and 0.108 ng of DNA was included in each PCR run as the reference standard. The quantity of targeted templates in each sample was determined relative to the reference DNA sample by the maximum second derivative method in the Roche LC480 program. The reaction was carried out in a Roche LightCycler 480 in 384-well plates, with triplicate wells for each sample. Dixon Q test was used to exclude outliners from the triplicates. The average of the T and S triplicate wells after outliner removal was used to calculate the T/S ratio for each sample. The same reference DNA was used for all PCR runs. The PCR efficiencies of the T and S reactions were 88.4% ± 4.1% and 91.4% ± 3.9% respectively.

The thermal profile for telomere (T) consisted of denaturing at 96ºC for 1 min followed by 30 cycles of denaturing at 96ºC for 1 s and annealing or extension at 54ºC for 60 s with fluorescence data collection. The thermal profile for a single copy gene (S) consisted of denaturing at 96ºC for 1 min followed by 8 cycles of denaturing at 95ºC for 15 s, annealing at 58ºC for 1 s, and extension at 72ºC for 20 s, followed by 35 cycles of denaturing at 96ºC for 1 s, annealing at 58ºC for 1 s, extension at 72ºC for 20 s, and holding at 83ºC for 5 s with data collection. We adapted the assay from the Cawthon 2002 study [15, 38]. The average inter-assay coefficient of variation (CV) for this study was 2.3 ± 1.5%. Intra-class correlation (ICC) of repeat extractions of 46 dried blood spot samples from this study was 0.883 (CI [0.766–0.942]).

### Statistical analysis

#### Maternal/parental characteristics

We evaluated whether the LTL data was normally distributed using a QQ plot graphical plot which confirmed LTL normal distribution. We conducted bivariate analyses using t-tests to evaluate the association between maternal and child LTL and dichotomous parental characteristics such as maternal high school education, Latinx parental ethnicity, paternal age (≤ 35 years and > 35 years), maternal smoking (yes/no), maternal mental illness pre-gestation or during pregnancy, maternal pre-existing or gestational hypertension, age of menstruation onset (≤ 12 years and > 12 years), and weight gain during pregnancy (≤ 40 lbs and > 40 lbs).

Parental categorical variables that were assessed in relation to child or maternal LTL included maternal education, marital status, parental race, maternal age (< 20, 20–25, 25–30, 30–35, 35–40, and > 40), maternal diabetes (preexisting diabetes, gestational diabetes, no diabetes), sugar-sweetened beverage (SSB) consumption (no SSB consumption, some (0–4/week) SSB consumption, high (> 4/week) SSB consumption), pre-pregnancy maternal body mass index (BMI) (underweight/normal, overweight, and obese), American College of Obstetrics and Gynecology (ACOG) [3] weight gain during pregnancy recommendations per pre-pregnancy BMI (under range [< 28 lbs (divide by 2.205 for kilograms) for < 18.5 BMI, < 25 lbs for 18.5–24.9 BMI, < 15 lbs for 25–29.9 BMI, and < 11 lbs for >=30 BMI], normal range [28–40 lbs for < 18.5 BMI, 25–35 lbs for 18.5–24.9 BMI, 15–25 lbs for 25–29.9 BMI, 11–20 lbs for >=30 BMI], and over range [> 40 lbs for < 18.5 BMI, > 35 lbs for 18.5–24.9 BMI, > 25 lbs for 25–29.9 BMI, and > 20 lbs for >=30 BMI]) [3], parity (1, 2/3, and > 3). Categorical variables were assessed for significance with maternal and child LTL using one-way analysis of variance tests. We also evaluated the relationship between weight gain within or outside ACOG limits and possible interaction with infant sex to determine infant LTL using linear regression models.

#### Child characteristics

Dichotomous child variables included gender (M/F), Apgar at 5 min (< 9, ≥ 9), birth type (vaginal, C-section), child birth weight Z-score (< 0, ≥ 0), child birth length Z-score (< 0, ≥ 0), and head circumference (≤ 34.5 cm, > 34.5 cm). Student t-tests were used to ascertain child and maternal LTL in relation to dichotomous variables. Similarly, categorical child characteristics such as gestational age (< 35 weeks, ≥ 35 and < 37 weeks, and ≥ 37 weeks), Apgar at 1 min (< 7, ≥ 7 and < 9, and ≥ 9), and child birth weight (all birth weights, low birth weight (<2500 grams), macrosomic (>4000 grams)) were assessed using one-way analysis of variance tests.

### Continuous variables

#### Maternal characteristics

Linear regression and Pearson’s correlation coefficient was used to assess the relationship between continuous variables such maternal LTL and the following continuous variables: maternal age, SSB intake, pre-pregnancy BMI, weight gain in pregnancy (lbs), age of menstruation onset, parity, gestational age, child birth weight (grams), birth weight Z-score, birth length Z-score, Apgar score at 1 min, Apgar score at 5 min, and head circumference (cm).

#### Child characteristics

Regressions and Pearson’s correlation coefficient was also performed to assess the association between child LTL and the variables mentioned above.

#### Multivariable linear regression

Multivariable linear regression models were used to assess independent predictors of maternal LTL and child LTL. Variables with statistical significance of *p* < 0.10 in bivariate analyses or potential biological plausibility were included in multivariable models.

We constructed two multivariable models of child LTL. One larger model (*n* = 351) included birthweight Z score, SSB weekly consumption, maternal Latina ethnicity, maternal age at menarche, gestational age, pre-existing or gestational mental illness and ACOG weight gain during pregnancy (within, above or below guidelines) and maternal pre-pregnancy BMI. A smaller model (*n* = 151) included paternal Latino ethnicity in addition to the aforementioned variables (excluding maternal pre-pregnancy and BMI to maximize sample size). The second model was significantly smaller as there was a sizable percentage of missing data for the second model as fewer families provided ethnicity information on fathers. We decided to run this smaller model to evaluate the potential role of paternal ethnicity on infant LTL.

In the maternal LTL model (*n* = 54), we included the continuous variables parity, SSB consumption per week, maternal age, maternal pre-pregnancy BMI, gestational weight gain in pregnancy and the dichotomous variable maternal high school education (high school diploma/no high school diploma).

## Results

### Predictors of child leukocyte telomere length

A total of 389 infants had a mean LTL of 2.02 ± 0.30 T/S (Table 1). We did not find any significant maternal or paternal level characteristics including maternal or paternal racial background, ethnicity or age or associated with newborn LTL (Table 1). However, fathers with Mexican/Central American ethnicity had shorter LTL (1.99 ± 0.25) than those with South American/Caribbean/Spain/Portuguese background (2.12 ± 0.40 T/S) (*P* = 0.02; Table 1).

**Table 1:** Parental demographics, health history, and birth metrics in relation to neonatal leukocyte telomere length (LTL)

Variable	Number (%) or Mean+/-SD	Mean T/S Ratio ±	*p* value	Pearson R (*p* value)
		Standard Deviation (SD)		
**Total Sample**	393	2.02 ± 0.30		
**Maternal and Paternal Demographics**				
*Maternal Education*				
Less than high school	53 (13.73)	2.03 ± 0.24	0.87	
High school graduate or more	333 (86.27)	2.02 ± 0.30		
*Maternal Education*				
Less than high school	53 (14.06)	2.03 ± 0.24	0.70	
High School graduate/GED only	126 (33.42)	2.00 ± -0.31		
Bachelor's/Associate's degree	116 (30.77)	2.04 ± 0.33		
Graduate degree	82 (21.75)	2.04 ± 0.27		
*Marital Status*				
Married	248 (64.42)	2.01 ± 0.28	0.23	
Single/Other	28 (7.27)	1.96 ± 0.21		
Living with Partner	109 (28.31)	2.06 ± 0.35		
*Maternal Latina*				
Latina	263 (66.92)	2.01 ± 0.28	0.51	
Other	130. (33.08)	2.04 ± 0.33		
*Maternal Latina Ethnicity*				
Mexican/Central				
American	195 (74.14)	2.01 ± 0.29	0.76	
South American(SA)/ Caribbean/Spain/ Portugal	68 (25.86)	2.01 ± 0.27		
*Paternal Latino Ethnicity*				
Mexican/Central American	138 (80.23)	2.00 ± 0.25	**0.02**	
Caribbean/Spain/Portugal	34 (19.77)	2.12 ± 0.40		
*Maternal Age (years)*	32.55 ± 5.44			-0.04 (0.48)
< 20	11 (2.86)	2.15 ± -0.27	0.44	
20–25	35 (9.11)	1.99 ± -0.23		
25–30	57 (14.84)	1.99 ± 0.28		
30–35	141 (36.72)	2.03 ± 0.25		
35–40	117 (30.47)	2.04 ± 0.24		
> 40	23 (5.99)	2.00 ± 0.27		
*Paternal Age (years)*	33.92 ± 6.65			0.01 (0.90)
≤ 35 years	135 (54.88)	2.01 ± 0.36	0.66	
> 35 years	111 (45.12)	2.02 ± 0.23		
**Maternal Health History and Lifestyle Characteristics**				
*Maternal Diabetes*				
Preexisting Diabetes (Type1/Type2/diet managed)	21 (5.34)	2.02 ± 0.26	0.32	
Gestational Diabetes	55 (14.00)	2.08 ± 0.38		
No Diabetes	317 (80.66)	2.01 ± 0.28		
*Smoking*				
Any smoking	40 (10.39)	2.02 ± 0.28	0.87	
No smoking	345 (89.61)	2.03 ± 0.30		
*SSB Consumption (servings/week)*	2.72 ± 5.39			-0.04 (0.48)
No SSB Consumption (= 0/week)	143 (36.76)	2.03 ± 0.32	0.69	
Some SSB Consumption (0–4/week)	171 (43.96)	2.01 ± 0.29		
High SSB Consumption (> 4/week)	75 (19.28)	2.01 ± 0.27		
*Maternal Mental Health*				
No Mental Health Incidence	332 (86.23)	2.03 ± 0.30	**0.09**	
Mental Illness Incident	53 (13.77)	1.96 ± 0.28		
*Pre-Pregnancy Maternal BMI Category*	26.13 ± 6.76			
Underweight/Normal (< 24.99)	198 (55.15)	2.01 ± 0.31	0.81	0.04 (0.48)
Overweight(25.0–29.99)	84 (23.40)	2.03 ± 0.32		
Obese(>=30)	77 (21.45)	2.03 ± 0.23		
*Maternal Hypertension*				
No hypertension present	328 (84.97)	2.02 ± 0.30	0.85	
Hypertension present	58 (15.03)	2.02 ± 0.26		
*Age of Menstruation Onset*	12.59 ± 1.61			
≤ 12 years	180 (46.88)	2.05 ± 0.32	0.16	-0.05 (0.36)
> 12 years	204 (53.12)	2.00 ± 0.28		
*Weight Gain during Pregnancy*	26.26 ± 13.13			0.04 (0.39)
< 40 lbs	339 (86.04)	2.01 ± 0.30	0.05	
> = 40 lbs	55 (13.96)	2.09 ± 0.30		
*Weight Gain during Pregnancy Within Guidelines for American College of Obstetrics and Gynecology (ACOG)*				
Under ACOG Range	86(22.99)	1.95 ± 0.24	**0.08**	
Normal ACOG Range	158 (42.25)	2.04 ± 0.34		
Above ACOG Range	130 (34.76)	2.03 ± 0.27		
*Parity*	1.83 ± 0.97			0.02 (0.71)
1	175 (54.18)	2.02 ± 0.30	0.67	
2,3	126 (39.01)	2.04 ± 0.31		
> 3	22 (6.81)	2.07 ± 0.29		
**Child Specific Variables**				
*Sex*				
Male	192 (49.74)	1.99 ± 0.30	**0.04**	
Female	194 (50.26)	2.06 ± 0.29		
*Neonatal Race/Ethnicity*				
European White	35 (8.91)	1.99 ± 0.21	0.09	
Asian (including Pacific Islander)	24 (6.11)	2.18 ± 0.51		
Black	3 (0.76)	1.99 ± 0.27		
Latinx	279 (70.99)	2.01 ± 0.28		
Missing/other	52 (13.23)	1.99 ± 0.28		
*Gestational Age*		38.90 ± 1.81		
< 35 weeks	16 (4.13)	2.15 ± 0.27	**0.04**	-0.12(0.02)
≥ 35 weeks and < 37 weeks	34 (8.79)	2.11 ± 0.44		
≥ 37 weeks	337 (87.08)	2.01 ± 0.28		
*Apgar (1 min)*		7.74 ± 1.32		0.05 (0.32)
< 7	46 (11.98)	1.99 ± 0.24	0.48	
≥ 7 and < 9	229 (59.63)	2.04 ± 0.29		
≥ 9	109 (28.39)	2.01 ± 0.33		
*Apgar (5 min)*		8.80 ± 0.62		0.01 (0.85)
< 9 at 5 min	55 (14.25)	1.98 ± 0.25	0.25	
≥ 9 at 5 min	331 (85.75)	2.03 ± 0.30		
*Birth Type*				
C-Section	94 (24.42)	2.02 ± 0.25	0.87	
Vaginal	291 (75.58)	2.02 ± 0.31		
*Child Weight (grams)*	3272.72 ± 536.37			-0.05 (0.30)
Low Birth Weight (LBW) (< 2500 g)	27 (6.98)	2.11 ± 0.27	0.11	
Macrosomic (> 4000 g)	26 (6.72)	1.99 ± 0.24	0.54	
*Child birth weight Z-score*	0.31 ± 0.99			0.91(-0.03)
< 0	239 (61.92)	2.02 ± 0.32	0.60	
≥ 0	147 (38.08)	2.03 ± 0.26		
*Child birth length Z-score*	0.21 ± 1.16			0.02 (0.71)
< 0	132 (34.38)	2.01 ± 0.33	0.40	
≥ 0	252 (65.62)	2.03 ± 0.28		
*Head Circumference (cm)*	34.17 ± 1.71			-0.09 (0.09)
≤ 34.5 cm	234 (60.78)	2.04 ± 0.30	0.13	
> 34.5 cm	151 (39.22)	2.00 ± 0.29		

Mothers with a history of mental illness had shorter infant LTL (1.96 ± 0.28 T/S) than mothers with no history (2.03 ± 0.30 T/S), although this relationship was not statistically significant (*P* = 0.086). Similarly, mothers who gained less than the ACOG recommended weight gain for pregnancy had infants with shorter LTL (1.95 ± 0.24 T/S) compared with mothers who gained the recommended or above the recommended amount (2.04 ± 0.34 T/S and 2.03 ± 0.27 T/S respectively; *P* = 0.08) although the difference did not meet statistical significance (Table 1). There was an overall trend of mothers who gained more weight having infants with longer LTL but the results were not statistically significant (> = 40 lbs versus < 40 lbs, 2.09 ± 0.03 T/S versus 2.01 +/-0.30) and neared statistical significance (*p* < 0.05).

We found few child level variables that were associated with longer LTL at birth (Table 1). Female infants had longer LTL than male infants (2.06 ± 0.29 T/S vs 1.99 + /0.30 T/S; *P* = 0.04) (Table 1) as did newborns with shorter gestational duration compared with newborns with longer gestation (r = -0.12, *P* = 0.02). Larger head circumference was associated with longer newborn LTL but the results trended towards statistical significance(r = -0.09, *P* = 0.09).

### Predictors of maternal leukocyte telomere length

For the 58 mothers with leukocyte telomere length (LTL) measured postnatally, mean LTL was 1.54 ± 0.26 T/S (Table 2). Older mothers tended to have shorter LTL than younger mothers (r = -0.30, *P* = 0.02) (Table 2). We did not find any association between other maternal or child variables and LTL including maternal demographics, health history and child anthropometrics at birth including infant LTL (Table 2).

**Table 2:** Maternal demographics, health history, and birth metrics in relation to maternal leukocyte telomere length (LTL)

**Variable**	N (%) or Mean+/-SD	Mean T/S Ratio ± SD	*p* value	Pearson R (*p* value)
**Total Sample**	58	1.53 ± 0.26		
**Maternal Demographics**				
*Maternal Latina Ethnicity*				
Mexican/Central American	39 (78.00%)	1.51 ± 0.25	0.23	
South America /Caribbean/Spain/Portugal	11 (19.30%)	1.63 ± 0.30		
* Maternal High School*				
Not high school graduate	16 (27.59%)	1.45 ± 0.16	0.10	
High school graduate	42 (72.41%)	1.58 ± 0.28		
* Maternal Education*				
Less than high school	16 (27.59%)	1.45 ± 0.16	0.15	
High school graduate/General Educational Development Test (GED) only	20 (34.48%)	1.62 ± 0.32		
Bachelor's/Associate's degree	22 (37.93%)	1.53 ± 0.24		
* Maternal Age*	32.78 ± 7.00		0.32	-0.30 (0.20)
≤ 35 years	39 (67.24%)	1.56 ± 0.28		
> 35 years	19 (32.76%)	1.49 ± 0.22		
* Marital Status*				
Married	32 (55.17%)	1.52 ± 0.21	0.72	
Single/Other	5 (8.62%)	1.53 ± 0.17		
Living with Partner	21 (36.21%)	1.58 ± 0.33		
**Maternal Health History**				
*Maternal Diabetes*				
Diabetes mellitus (including gestational)	11 (19.00%)	1.57 ± 0.34	0.73	
No diabetes mellitus (or gestational)	47 (81.00%)	1.53 ± 0.24		
* Smoking*				
Any smoking	8 (13.80%)	1.44 ± 0.16	0.26	
No smoking	50 (86.20%)	1.56 ± 0.27		
* Hypertension*				
Hypertension (including gestational)	9 (15.52%)	1.50 ± 0.19	0.61	
No hypertension (including gestational)	49 (84.48%)	1.55 ± 0.27		
* Sugar Sweetened Beverage (SSB) Intake*				
No SSB Consumption (= 0/week)	7 (12.07%)	1.52 ± 0.14	0.60	-0.10 (0.48)
Any SSB Consumption (> 0/week)	33 (56.90%)	1.57 ± 0.26		
High SSB Consumption (> 4/week)	18 (31.03%)	1.49 ± 0.28		
*Mental Health*				
No mental illness	45 (78.95%)	1.56 ± 0.27	0.37	
Mental illness	12 (21.05%)	1.48 ± 0.22		
* Pre-pregnancy Body Mass Index (BMI) kg/m*^*2*^	27.96 ± 8.44			0.06 (0.68)
Normal (< 25)	23 (39.66%)	1.56 ± 0.20	0.69	
Overweight (25–29.99)	20 (34.48%)	1.50 ± 0.25		
Obese (≥ 30)	15 (25.86%)	1.57 ± 0.33		
Weight Gain in Pregnancy, pounds	23.96 ± 16.82			0.14 (0.31)
≤ 40 lbs	52 (91.23%)	1.53 ± 0.27	0.40	
>40 lbs	5 (8.77%)	1.64+/-0.13		
* American College of Obstetrics and Gynecology (ACOG) Weight Gain during Pregnancy*				
Under ACOG Range	16 (29.63%)	1.57 ± 0.30	0.77	
Normal ACOG Range	20 (37.04%)	1.48 ± 0.20		
Above ACOG Range	18 (33.33%)	1.54 ± 0.29		
* Menarche (age, years)*	12.36 ± 1.55			-0.07(0.61)
≤ 12 years old	30 (53.57%)	1.57 ± 0.29	0.55	
> 12 years old	26 (46.43%)	1.53 ± 0.21		
* Parity*	2.02 ± 0.94			-0.04 (0.79)
1	20 (34.48%)	1.49 ± 0.21	0.44	
2,3	35 (60.34%)	1.58 ± 0.28		
> 3	3 (5.18%)	1.46 ± 0.22		
**Child Specifics**				
Maternal LTL correlated with Child LTL				0.03 (0.84)
*Gestational Age*	38.68 ± 1.89			-0.11(0.42)
< 37 weeks	51 (87.93%)	1.53 ± 0.26		
≥ 37 weeks	7 (12.07%)	1.65+/-0.24		
* Birth Weight Z-Score*	-0.31 ± 1.02			-0.03 (0.81)
< 0	38 (65.52%)	1.53 ± 0.23	0.73	
≥ 0	20 (34.48%)	1.56 ± 0.31		
* Birth Length Z-Score*	0.14 ± 1.20			-0.03(0.81)
< 0	21 (36.21%)	1.61 ± 0.26	0.15	
≥ 0	37 (63.79%)	1.50 ± 0.25		
* Apgar 1 min*	7.74 ± 1.32			
< 7	7 (12.07%)	1.58 ± 0.21	0.72	
≥ 7 and < 9	35 (60.34%)	1.52 ± 0.26		
≥ 9	16 (27.59%)	1.57 ± 0.27		
* Apgar at 5 min*	8.80 ± 0.62			-0.18(0.18)
< 9 at 5 min	12 (20.69%)	1.60 ± 0.22	0.38	
≥ 9 at 5 min	46 (79.31%)	1.52 ± 0.27		
* Head Circumference (cm)*	34.23 ± 1.67			-0.12(0.98)
≤ 34.5 cm	34 (58.62%)	1.54 ± 0.26	0.35	
> 34.5 cm	24 (41.37%)	1.54 ± 0.26		

### Multivariable predictors of newborn leukocyte telomere length

**Fig. 1:**
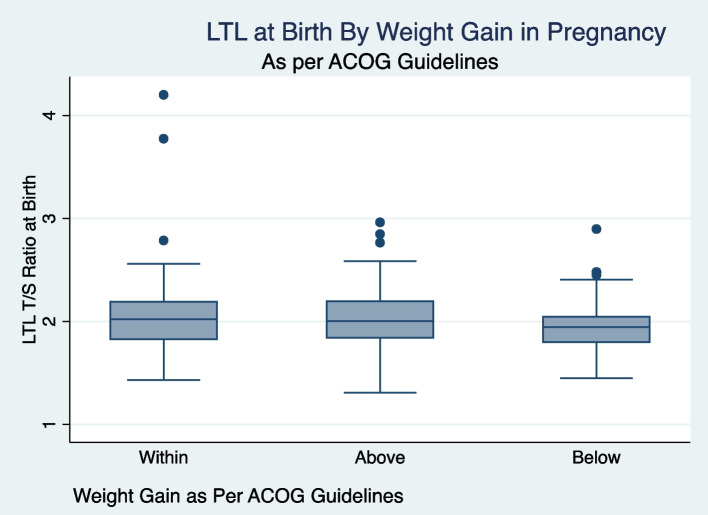
LTL at birth by ACOG weight gain category in pregnancy

**Fig. 2:**
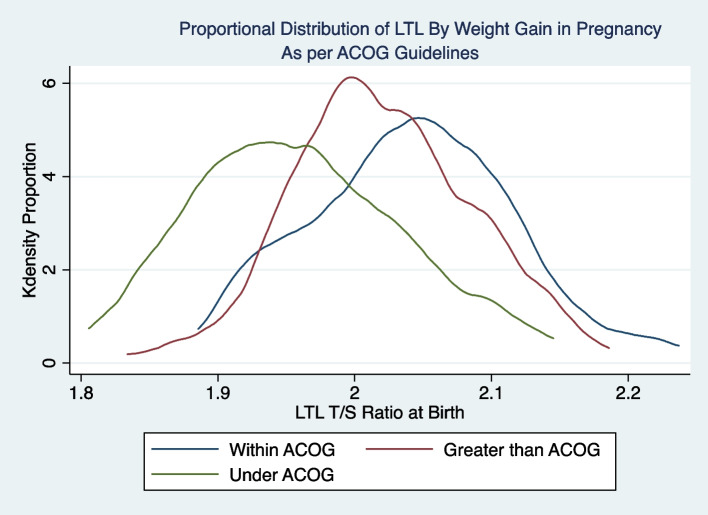
Proportional distribution of LTL by ACOG weight gain category in pregnancy

In multivariable model (*N* = 359) of newborn LTL including maternal Latina ethnicity, maternal mental illness during pregnancy, age of menarche, maternal pre-pregnancy BMI, weight gain in pregnancy based on ACOG recommendations, maternal diabetes mellitus (pre-existing or gestational), newborn sex, gestational age, birth weight Z-score, and maternal SSB consumption per week, three variables were associated with newborn LTL (Table 3). Female sex was associated with significantly longer newborn LTL compared with male (ß-coefficient = 0.08, 95%CI 0.02–0.14; *p* = 0.01). Longer gestational duration compared with shorter gestation was associated with shorter LTL (ß-coefficient = -0.03, -0.06-(-)0.01; *p* < 0.01). Mothers with weight gain below the ACOG recommendation also had newborns with shorter LTL (ß-coefficient = -0.10, (95%CI -0.18–0.02; *p* = 0.02). (Table 3; Figs. 1 and 2). We evaluated the possible role of interaction between gestational weight gain per ACOG recommendations and infant sex in relation to infant LTL not finding any significant statistical effect for the interaction term.

**Table 3:** Predictor of neonatal Leukocyte Telomere Length (LTL)

Variable Name	Beta Coefficient (95% Confidence Interval)	*P* Value
Latina Ethnicity	0.04(-0.03–0.008)	0.23
Age of Menarche, years	-0.01 (-0.03–0.008)	0.27
Weight gain in pregnancy		
Above ACOG^a^ Guidelines	0.002 (-0.07–0.07)	0.96
Below ACOG^a^ Guidelines	**-**0.10 (-0.18-(-)0.02)	**0.02**
Gestational age, weeks	-0.03 (-0.05-(-)0.01)	** < 0.01**
Female Sex	0.08 (0.02–0.14)	**0.01**
Birthweight Z Score	0.009 (-0.03–0.05)	0.66
SSB^b^ Serving Per Week	-0.001 (-0.008–0.005)	0.63
Pre-pregnancy body mass index (kg/m2)	0.0002 (-0.0048–0.005)	0.95
Mental illness in pregnancy	-0.05 (-0.14–0.04)	0.25

^a^American College of Obstetrics and Gynecology (ACOG)

^b^Sugar sweetened beverage (SSB)

In a smaller model of newborn LTL including paternal Latino ethnicity categories (Mexican/Central American versus South American/Caribbean/Spain/Portuguese), maternal age, parity, maternal education, weight gain within, below or above ACOG recommendations and pre-pregnancy BMI with a sample size (*N* = 156), two variables were associated with shorter newborn LTL. Increased gestational age was associated with shorter LTL (ß = coefficient = -0.04, -0.07-(-)0.01; *p* < 0.01) and mothers with weight gain below the ACOG recommendation similarly were associated with shorter newborn LTL (ß-coefficient = -0.15,95%CI -0.26-(-)0.04; *p* < 0.01) (results not shown). Paternal Latino ethnicity of South American/Caribbean/Spanish/Portuguese origin was associated with longer LTL in newborns but the results trended to statistical significance (ß-coefficient = 0.11, 95%CI -0.001–0.23; *p* = 0.07) (results not shown). Similar to the above larger model of newborn LTL, male sex was associated with shorter LTL compared with female newborns although the results trended towards statistical significance (Coeff -0.08, 95%CI -0.17–0.008, *P* = 0.07).

### Multivariable predictors of maternal leukocyte telomere length

In a model of maternal LTL (*n* = 54) including maternal parity, SSB weekly consumption (servings), maternal age, education level and pre-pregnancy BMI, increasing maternal age was associated with a decreased maternal postnatal LTL (ß-coefficient = -0.02, 95%CI -0.03—0.005) (Table 4). No high school diploma was associated with shorter maternal LTL compared with having a high school diploma (ß- coefficient = 0.18, 95%CI 0.003–0.36; *p* = 0.046) (Table 4).

**Table 4:** Predictors of maternal leukocyte telomere length (LTL)

Variable Name	Beta Coefficient (95% Confidence Interval)	*P* Value
Maternal Age, years	-0.02 (-0.03–0.005)	** < 0.01**
Parity	0.06 (-0.03–0.14)	0.20
High School Diploma	0.18 (0.003–0.36)	**0.046**
Maternal Pre-pregnancy BMI (kg/m2)	-0.0003 (-0.009–0.009)	0.96
Weight Gain in Pregnancy (pounds)	-0.002 (-0.007–0.002)	0.30

## Discussion

### Inadequate gestational gain and shorter LTL

Our data from a primarily Latina cohort indicate that sub-optimal maternal weight gain during pregnancy (using definitions from ACOG) is associated with a reduction in newborn infant LTL. We found the longest infant LTL to be from the normal weight gain category, providing evidence at the cellular level of the integrity of these ACOG recommendations. Our studies confirm findings from another group that found shorter fetal LTL with inadequate gestational weight gain earlier in gestation [46]. Maugeri and colleagues similarly utilized a longitudinal birth cohort design, but assessed fetal LTL in cell-free DNA from amniotic fluid at a mean of 16 weeks, in a European Italian population. Another European study (Belgian cohort) however did not find any association between weight gain in pregnancy, similarly using the adequate/insufficient or excessive weight gain based on ACOG categories [42]. This study, however, had a much lower percentage of women with pre-pregnancy overweight or obesity compared with ours (34.3% in the European cohort (10.7% obese) versus 44.85% in our cohort including 21.45% obese). We also had a relatively high percentage of women who had insufficient gain in pregnancy (25%), and it is not clear how many women in the Belgian cohort did not have adequate gain. It is possible that demographic and health characteristics differences between studies explain disparate findings.

In addition to our observations of negative LTL effects from inadequate gestational weight gain, other adverse maternal and neonatal outcomes are related to high and low gestational weight gain [29], and appear to follow a U-shaped curve [67]. Although not statistically significant, there was a trend in our data also of shorter LTL with excessive gestational gain. Among Latina mothers, in particular, disparate gestational weight gain and aforementioned outcomes exist, and are closely associated with pre-pregnancy BMI [19], so future steps, such as nutrition counselling, are essential in reducing adverse infant outcomes. Previous studies have found that inadequate and excessive gestational gain are associated with epigenetic changes including DNA methylation in the placental and umbilical cord tissues [35, 44]. The temporal relationship between maternal phenotypic traits such as diet or weight gain in pregnancy, DNA methylation patterns and infant LTL are not clear although one study has suggested that epigenetic changes may mediate the relationship between maternal phenotypes and infant LTL [6].

### Maternal education level and maternal/child LTL

Maternal educational disparities often track with health outcomes (both for mothers and children), as education level is a good proxy of overall socioeconomic status [56]. In our sample, we found that high school education was significantly associated with longer maternal LTL, which aligns closely with past findings [1, 4, 40]. When stratifying our high school educated mothers further, however, we did not find any significant differences between college educated vs high school educated mothers compared to those who had education less than high school, similar to recent findings [4]. This adds to growing literature that educational attainment is an important determinant of LTL in adult life, potentially due to the role that educational attainment plays in buffering against stress and as a correlate of socioeconomic status [60, 62]. We did not find, however, any association between maternal education level and infant LTL in contrast with our previous findings [69].

###  Infant sex and LTL

Similar to previous findings in Latinx newborns [69], in this larger cohort of primarily Latinx newborns, we found significantly longer newborn LTL in females compared to males, a finding that has been explored in recent studies (Bosquet [23, 24, 26, 33] but not replicated. One other study reported a greater difference between males and females [16] than our findings. We had a 3.40% difference between male and female LTL T/S ratios compared with a 4.39% difference in a large study of Chinese, Malay and Indian infants in a Singaporean cohort [16]. Our previous finding in cord blood in a smaller sample (*n* = 54) had a 5.1% difference between male and female newborns [69]. Other US based studies have not found statistically significant differences between male and female LTL at birth [23, 53] or a smaller difference than our finding (1.46%) [25] and a study of infants at 3 months of age from the Netherlands also did not find any difference between girls and boys [26]. It is possible that there is interaction between racial/ethnic and sex differences in determining newborn LTL at birth as some studies with white and black newborns have found [20] more pronounced differences between certain racial/ethnic groups.

### Parental demographics and infant LTL

Previous studies have demonstrated significant differences in racial background and LTL in infants [20, 25, 49]. In particular, populations with African/black origin have longer LTL than other population groups [30, 32, 40, 52].

As Latinx ethnicity comprises heterogeneous population groups with different racial backgrounds, we sought to further categorize place of origin within our diverse cohort of Latinx families. We found longer LTL of infants from fathers with Caribbean, South American, Spanish or Portuguese backgrounds although the results only trended to statistical significance. It is possible that this group reflects the African ancestry of the Caribbean origin fathers, who comprised about 20% of our Latinx fathers. Black African and African-Americans have longer LTL than non-Hispanic whites in many other studies in adults [49, 57, 65], as well as infants [13, 20, 25]. This is the first study to report on differences in infant LTL within a Latinx cohort on the basis of geographic origin. Interestingly, this study is also one of the few studies to delineate differences in LTL on the basis of paternal geographic origin. As our sample that included data on paternal ethnicity and race was smaller, future studies should evaluate the role of maternal and paternal ethnic and racial background on infant LTL.

### Negative findings

Overall, our study did not replicate the association between adverse maternal health exposures in pregnancy and shorter LTL in neonates, possibly due to the relative homogeneity of our sample in terms of socio-demographics and the absence of common chronic health conditions.

Other studies have found that maternal stress during pregnancy is associated with decreased maternal LTL [47] and infant LTL [58]. We did find that pre-existing or gestational mental illness had a negative impact on child LTL in univariate analysis, although results did not meet statistical significance in multivariable models, which may have been the result of a small sample size of patients with recorded mental illness and a lack of data on severity or duration. We had approximately 14% of mothers report mental health issues in pregnancy and we did not differentiate between incident diagnosis or pre-existing conditions. We also did not collect data on treatment or remission and it is possible that untreated mental illness may have more adverse impacts on neonatal LTL. Previous studies have found that maternal stress in pregnancy is associated with shorter LTL [58]. Alternatively, the impact on child LTL may be delayed and exposure to maternal mental illness in utero may only impact child health at later timepoints as other studies have indicated with school age children [61].

We also did not find any association with SSB consumption in pregnancy and child or maternal LTL. From previous studies, including some of our own, sugar-sweetened beverage (SSB) consumption has been shown to be associated with shorter LTL in early childhood, adolescence, and adulthood [10, 37, 68] however few studies have been conducted with pregnant women. A previous study with longitudinal LTL measurements found that pregnant women with reduced SSB consumption had decreased LTL attrition over time but did not find any association between LTL and overall SSB consumption. Our sample size of LTL in postpartum women in this study was small and it is possible that impacts on children may also be delayed and further follow-up is warranted.

We also did not find any association between parental age and infant LTL. Other studies have shown positive associations between paternal age and children’s LTL [8, 12, 16, 31, 71], and slight negative correlations with advancing maternal age [21]. However, our paternal and maternal age distribution was narrow. In the case of our fathers, the majority were under the age of 35, where advanced paternal age has been shown to correlate with increased infant LTL in previous studies [16, 31].

### Future directions

Future studies could evaluate the role of maternal gestational gain on telomere length in other tissue types such as placental and umbilical cord that could better elucidate pathways between maternal health in pregnancy and neonatal outcomes. Longitudinal measurements of placental LTL could help elucidate whether gestational gain at certain time periods in pregnancy were of particular significance. Other studies have suggested that DNA methylations patterns may mediate the relationship between parental phenotypes and infant LTL and as such future infant LTL studies could also include evaluation of DNA methylation [5]. In this study, we did not collect paternal LTL data, which may have been helpful in substantiating some of the associations we found between paternal variables and child LTL. Our study was also limited by sample size in the analysis of health variables that had a low frequency in our population, including smoking during pregnancy, pre-existing diabetes mellitus and hypertension.

## Data Availability

Data is available by email request and review from the principal investigator of the study (Wojcicki).
